# 

**Published:** 1871-09

**Authors:** 


### CONTENTS.

COMMUNICATIONS.

Oxygen in its Relationship to the Vital Phenomena ..... 369
Dentistry *vs.* Money ..... 379
Valedictory Address ..... 387
Dental Jottings ..... 391

PROCEEDINGS OF SOCIETIES.

Minutes of the 13th Annual Meeting of the Indiana State Dental Association ..... 394
Extracts from Proceedings of the Forest City Society of Dental Surgeons, Cleveland, Ohio, June 6th, 1871 ..... 400

EDITORIAL.

Crude Teachings ..... 406
Examination ..... 407
Wisconsin State Dental Society ..... 409
American Dental Association ..... 411
Obituary ..... 412

